# Cells with stochastically increased methyltransferase to restriction endonuclease ratio provide an entry for bacteriophage into protected cell population

**DOI:** 10.1093/nar/gkac1124

**Published:** 2022-12-08

**Authors:** Alexander Kirillov, Natalia Morozova, Svetlana Kozlova, Vasilisa Polinovskaya, Sergey Smirnov, Mikhail Khodorkovskii, Lanying Zeng, Yaroslav Ispolatov, Konstantin Severinov

**Affiliations:** Skolkovo Institute of Science and Technology, Center for Molecular and Cellular Biology, Moscow 121205, Russia; Peter the Great St. Petersburg Polytechnic University, St. Petersburg 195251, Russia; Skolkovo Institute of Science and Technology, Center for Molecular and Cellular Biology, Moscow 121205, Russia; Peter the Great St. Petersburg Polytechnic University, St. Petersburg 195251, Russia; Skolkovo Institute of Science and Technology, Center for Molecular and Cellular Biology, Moscow 121205, Russia; Skolkovo Institute of Science and Technology, Center for Molecular and Cellular Biology, Moscow 121205, Russia; Skolkovo Institute of Science and Technology, Center for Molecular and Cellular Biology, Moscow 121205, Russia; Peter the Great St. Petersburg Polytechnic University, St. Petersburg 195251, Russia; Texas A&M University, Department of Biochemistry and Biophysics, Center for Phage Technology, College Station, TX 77843, USA; University of Santiago of Chile (USACH), Physics Department, Av. Víctor Jara 3493, Santiago, Chile; Waksman Institute for Microbiology and Department of Molecular Biology and Biochemistry, Rutgers, State University of New Jersey, Piscataway, NJ 08854, USA

## Abstract

The action of Type II restriction–modification (RM) systems depends on restriction endonuclease (REase), which cleaves foreign DNA at specific sites, and methyltransferase (MTase), which protects host genome from restriction by methylating the same sites. We here show that protection from phage infection increases as the copy number of plasmids carrying the Type II RM Esp1396I system is increased. However, since increased plasmid copy number leads to both increased absolute intracellular RM enzyme levels and to a decreased MTase/REase ratio, it is impossible to determine which factor determines resistance/susceptibility to infection. By controlled expression of individual Esp1396I MTase or REase genes in cells carrying the Esp1396I system, we show that a shift in the MTase to REase ratio caused by overproduction of MTase or REase leads, respectively, to decreased or increased protection from infection. Consistently, due to stochastic variation of MTase and REase amount in individual cells, bacterial cells that are productively infected by bacteriophage have significantly higher MTase to REase ratios than cells that ward off the infection. Our results suggest that cells with transiently increased MTase to REase ratio at the time of infection serve as entry points for unmodified phage DNA into protected bacterial populations.

## INTRODUCTION

In nature, bacteria are constantly under attack from viruses (bacteriophages) (1). Multiple mechanisms of bacterial defense against viral infection have evolved. Abortive infection mechanisms kill infected cells before viral progeny can form (2). Such altruistic behavior benefits clonal population limiting the spread of the virus. The CRISPR-Cas systems record and target specific phages that a bacterial cell or its ancestors have previously encountered and thus provide adaptive immunity (3). Another common resistance mechanism is provided by restriction–modification (RM) systems (4–6). These systems protect cells from diverse, unrelated phages and thus can be considered as a form of innate immunity. While several mechanistically distinct types of RM systems have been described, the Type II is the most common (4). The defensive action of these systems is due to activity of two enzymes, a restriction endonuclease (REase) and a cognate methyltransferase (MTase). Both enzymes recognize identical, usually 4–6 bp, sites in the DNA. An REase cleaves (restricts) DNA at or close to unmodified sites, while the cognate MTase methylates (modifies) specific bases in the recognized sites. Because most recognition sites are palindromic, MTases introduce two methyl groups at both strands of the recognized sequence, generating first hemimethylated and then fully methylated sites, neither of which are recognized by cognate REases and are thus not subject to restriction (7–9). The RM systems clearly impact on phages, which respond to this pressure in multiple ways. In laboratory settings, decreasing the number of recognition sites in the genome increases the ability of phage to overcome RM protection of the host (10,11). Consistently, partial or complete avoidance of host RM systems recognition sites in phage genomes, presumably acquired in the course of evolution, has been demonstrated (12). Other mechanisms include phage encoded diverse anti-restriction proteins, extensive modification of bases in phage DNA, or complete substitution of standard DNA bases for non-standard ones, for example, using uracils instead of thymines (13–17).

While the presence of an RM system in a bacterial host provides strong protection from attack by sensitive phages (18), this protection is short-lived. Progeny phages with modified DNA invariably arise at least at conditions of laboratory experiments. Once such epigenetically modified phages appear, they productively infect cells with a cognate RM system, rendering the defense useless. On the other hand, when the modified phage infects a cell without an RM system, unmodified phage progeny is released and the initial level of protection is restored.

The intracellular amounts/activity levels of the MTase and REase enzymes shall be tightly controlled because an imbalance can either lead to the death of uninfected (excess REase activity) (19,20) or infected (excess MTase activity) cells (20). Indeed, diverse regulatory mechanisms that ensure the amounts of RM enzymes are tightly controlled have been described (reviewed in (21)). Known mechanisms include antisense RNAs that target RM genes transcripts (22,23), MTases that bind specific operator sequences located in front of RM genes promoters (24–26), and C proteins, DNA binding transcription factors encoded within some RM gene clusters. By binding to multiple operator sites located in front of RM genes promoters, C proteins ensure optimal levels of transcription of the RM genes and also homeostatically regulate the transcription of their own gene (27–31). This type of regulation, first reported for the PvuII system (32), appears to be very widespread (33).

Though the above mentioned mechanisms, as well as others, yet to be discovered, ensure homeostasis of RM gene expression through multiple positive and negative feedback loops, the fact that RM protection can be overcome with appreciable (∼10^−3^) frequency suggests that in some infected cells at least hemimethylation of each of the recognition sites in the infecting phage genome must occur before even a single cleavage event by the REase takes place. In principle, this can happen when activities of the MTase and REase enzymes in an infected cell are altered such that excessive MTase activity allows preemptive modification of phage DNA.

Earlier, we used mathematical modeling to show how the ability of a phage to overcome protection afforded by an RM system can be determined by the relative ratio of MTase and REase activities in the cell (34). This result seems to be collaborated by recent experimental analysis, where it was shown that the level of protection afforded by a plasmid-borne EcoRI system is not directly related to the absolute amounts of RM enzymes and, in fact, can be even negatively correlated with RM enzymes abundance (20). To further verify the model predictions we here experimentally perturbed the intracellular amounts of MTase and REase of C protein-dependent RM system Esp1396I (30,35). Similarly to other C protein-dependent RM systems, Esp1396I C protein finely controls the steady-state amounts of Esp1396I MTase and REase present inside the cell through multiple interactions with operator sites located in front of the Esp1396I MTase gene and its own gene, which is co-transcribed with the REase gene (30). This regulation ensures coordinated synthesis of both RM enzymes when a plasmid containing the Esp1396I system enters a naïve cell (36). In agreement with earlier theoretical analysis, we show that changing the ratio of MTase and REase enzymes while keeping the intracellular concentration of one of the enzymes constant can have a drastic effect on the level of protection from phage infection. High MTase to REase ratios abolish protection, while lowering the MTase to REase ratio makes it impossible for the phage to initiate productive infection. We also determined the effects of increasing the absolute amounts of individual Esp1396I enzymes on the half-life of unmodified phage DNA inside the cell and showed that the rate of infecting DNA degradation is affected by MTase to REase ratio. By examining the levels of stochastic variation of MTase and REase enzymes’ amounts in individual cells before and after phage infection, we show that cells with increased MTase to REase ratio at the time of the infection serve as entry points for unmodified phage into protected bacterial populations.

## MATERIALS AND METHODS

### Bacterial strains, phage, and plasmids

The *E. coli* strains, phage, and plasmids used in this work are listed in Supplementary Table S1. *Escherichia coli* $\rm {DH5\alpha}$ was used for cloning, flow cytometry, and most microscopy experiments. *E. coli* K-12 MG1655 cells encoding the SeqA protein fused with the mKO2 orange fluorescent protein (37) were used to observe bacteriophage λ_vir_ DNA degradation. *E. coli* Rosetta cells were used for Venus and mCherry fluorescent proteins overproduction. Bacteriophage λ_vir_ (38) was used throughout.

pET21a-based plasmids expressing Venus or mCherry genes, as well as the pUC-based plasmid expressing the fluorescently-labeled Esp1396I*_*fluo RM system (pRM-High) were described previously (36).

To create the pRM-Mid plasmid, the entire fluorescently-labeled Esp1396I*_*fluo RM system was amplified from pRM-High with primers ‘RM-Mid_for’ and ‘RM-Mid_rev’ (Supplementary Table S1) containing, respectively, EcoRI and HindIII restriction sites introduced for cloning. The pBR322 vector was linearized using EcoRI and HindIII and used to clone EcoRI/HindIII treated PCR fragment containing fluorescently-labeled Esp1396I*_*fluo RM system.

To create the pRM-Low plasmid, a fragment of pRM-High containing the entire fluorescently-labeled Esp1396I*_*fluo RM system and the adjacent *bla* (ampicillin resistance) gene was amplified with ‘pRM_for’ and ‘pRM_rev’ primers (Supplementary Table S1). A fragment containing the pSC101 origin was amplified using the ‘pSC101_ori_F’ and ‘pSC101_ori_R’ primers (Supplementary Table S1). The two amplified fragments were combined using Gibson assembly (39) to generate pRM-Low.

To create plasmids for individual expression of R and M fusions, the MTase::Venus and REase::mCherry fused genes from pRM-High were amplified, respectively, with ‘M::Venus_for’/‘M::Venus_rev’ and ‘R::mCherry_for’/‘R::mCherry_rev’ primer pairs. The forward primers contained an NdeI site (CATATG, with ATG overlapping with the first codon of amplified *esp1396I* genes), the reverse primers contained a KpnI site downstream of amplified fusion genes stop codons. After NdeI/KpnI digestion, amplified fragments were cloned downstream of the *araBAD* promoter of the pBAD/HisB plasmid (Invitrogen) linearized with NdeI and KpnI. As a final step, plasmid pACYC184 was amplified with primers ‘pACYC_for’ and ‘pACYC_rev’ (Supplementary Table S1). The primers contain sequences matching the *araBAD* promoter. The MTase::Venus or REase::mCherry genes cloned in pBAD/HisB were amplified with primers ‘araBAD_for’ and ‘araBAD_rev’ (Supplementary Table S1) containing sequences overlapping with pACYC184. Gibson Assembly was used to combine amplified pACYC184 and MTase::Venus or REase::mCherry genes. The resulting plasmids were named pACYC_M_fluo (or ‘+M’) and pACYC_R_fluo (or ‘+R’). All plasmids were sequenced to confirm the absence of mutations.

### Bacterial growth and phage lysate preparation

Unless otherwise stated, cells were grown in liquid of solid (1.5% agar) LB medium (Amresco). Where appropriate, antibiotics were added at concentrations of $100\,{\rm \mu g/ml}$ (ampicillin) and $25\,{\rm \mu g/ml}$ (chloramphenicol). For plasmid transformation, competent cells were prepared as described (40). Cells carrying two compatible plasmids were obtained by co-transformation and selection of transformed colonies on media containing two antibiotics. Expression of MTase or REase fusions from the *araBAD* promoter was performed by the addition of different concentrations (from 1 $\mu {\rm{M}}$ to 12 mM) of l-arabinose to growing cell cultures. To prevent leaky expression from the *araBAD* promoter, 0.2% glucose was added where indicated. Bacteriophage λ_vir_ lysates were prepared as described elsewhere (41).

### Plasmid copy number determination

Total DNA was extracted from bacterial exponentially growing cultures with GeneJET Genomic DNA Purification Kit (Thermo Scientific). Primers specific for the Esp1396I MTase gene (‘Esp1396I_meth_for’ and ‘Esp1396I_meth_rev’, Supplementary Table S1) and for the chromosomal *gyrA* gene (‘gyrA_for’ and ‘gyrA_rev’, Supplementary Table S1) were designed using the PrimerQuest Tool (Integrated DNA Technologies). qPCR reactions were performed in 10 μl mixtures using iTaq Universal SYBR Green supermix (Bio-Rad). Separate reactions were prepared for detection of chromosomal and plasmid-specific amplicons, and each reaction was carried out in triplicate. To calculate the absolute concentration of plasmid and genomic amplicons, efficiency of qPCR was estimated from standard curves obtained from qPCR reactions with aliquots of serial dilutions of solutions containing known concentrations of purified pRM-High or chromosomal DNA as templates. PCN was calculated by taking a ratio of absolute concentrations of plasmid and genomic DNA in the sample (the 2^−ΔΔ^^CT^method, (42)).

### Phage protection plaque assay

Overnight cultures of *E. coli* cells carrying various plasmids were grown in LB with the addition of ampicillin (pLow, pMid, pHigh, pRM-Low, pRM-Mid or pRM-High) or ampicillin and chloramphenicol (pRM-Mid + R or + M) at 37°C. Cells were diluted 1:100 in fresh LB medium with the addition of 10 mM MgSO_4_, 0.2% maltose and antibiotics, and grown at 37°C to OD_600_ ≈0.4. Petri dishes were precast with 10 ml of 1.5% LB agar (bottom layer) and then overlaid with 4 ml of 0.4% LB agar (top layer) mixed with the 100 μl of cell cultures, 100 μl of serial dilutions of bacteriophage lysate, 10 mM MgSO_4_, 0.2% maltose, and appropriate antibiotics. The plates were incubated for 10 h at 37°C and phage titers were determined as described in (43).

### Protein purification

Purified Venus and mCherry fluorescent proteins were used as standards for microscopy calibration (36). The Rosetta strain was transformed with the pET21a-based Venus or mCherry expression plasmids. Overnight cultures from the single colonies were grown in LB medium with the addition of ampicillin and chloramphenicol at 37°C. Cells were diluted 1:100 in 50 ml of fresh LB medium with the addition of ampicillin and chloramphenicol and were grown at 37°C till OD_600_ = 0.6. Protein expression was induced by the addition of 1 mM IPTG. After 4 h of growth, cells were centrifuged at 4000 g, and the pellet was resuspended in 50 mM Tris–HCl, 500 mM NaCl, 10 mM imidazole. Cells were lysed by sonication in the presence of lysozyme (1 mg/ml) and PMSF (1 mM). The Venus and mCherry proteins C-terminally tagged with a 6-His-tag were affinity-purified from the cleared cell lysate on a 1-ml Ni-NTA agarose column (Invitrogen) (44). After the loading of cell lysate onto the column, it was washed with three volumes of 50 mM Tris–HCl, 500 mM NaCl, 10 mM imidazole buffer, and protein was eluted using 50 mM Tris–HCl, 500 mM NaCl, 400 mM imidazole buffer. Fractions containing purified target proteins (∼95% pure as judged by visual inspection of SDS gels) were diluted with glycerol (50% final concentration), frozen in liquid nitrogen, and stored at –80°C prior to use. The concentration of proteins was measured using the Bradford assay (45).

### Fluorescence intensity calibration

Purified Venus and mCherry proteins with known concentrations were used as a standard for spectrofluorimetric (Varian Cary Eclipse) measurements from which the fluorescence of a single fluorescent protein molecule was calculated. Fresh cultures of *E. coli* Rosetta cells with pET-based Venus or mCherry expression plasmids were grown in LB medium with the addition of ampicillin and chloramphenicol and 1 mM IPTG. The concentration of cells was measured using live microscopy, and culture fluorescence was measured using Varian Cary Eclipse fluorescence spectrophotometer. This data allowed calculating the average fluorescence of a single cell and, consequently, the average number of fluorescent proteins per cell. Simultaneously, average fluorescence of a single cell observed by live microscopy was measured using ImageJ (Fiji) tools (46). The autofluorescence signal was calculated from data obtained with microscopy of cells harboring empty plasmid vectors. After subtraction of the mean autofluorescence signal, the fluorescence of a single fluorescent protein molecule under the microscope was calculated. This allowed us to determine the value of a coefficient for recalculation of fluorescence intensity of a cell to the number of fluorescent protein molecules present in it.

### Microscopy

Fluorescence microscopy was performed using a Nikon Eclipse Ti-E inverted microscope equipped with a custom incubation system. Fluorescence signals in Venus and mCherry fluorescence channels were detected using Semrock filter sets YFP-2427B and TxRed-4040C, respectively. The long-time microscope filming was performed using Micro-Manager 2.0 tools (47). Time-lapse filming was performed using sCMOS ZYLA 4.2MP Plus Andor camera. Image analysis was performed using ImageJ (Fiji) (46) with the Circle4px plugin used for measurements of fluorescence in individual cells.

The double-sided tape (Scotch) was used for the frame formation on one microscopic slide. The 1.5% agarose (Helicon) diluted in 0.25% LB was used to make a smooth surface inside the frame. The agarose pad was pressed by the second similar slide. After agarose solidification, the upper slide was removed. Thin agarose strips were cut to provide oxygen to the sample in the chamber. The sample mixture was put on the thin agarose pads. When the mixture was fully absorbed, the chamber was sealed with the coverslip (24 × 40 mm, Menzel-Gläser).

### Observation of bacteriophage λ_vir_ DNA inside bacterial cells

This was done as described in detail elsewhere (48). *E. coli* K-12 MG1655 cells encoding the SeqA protein fused with the mKO2 orange fluorescent protein were grown in M9 to OD_600_ = 0.4 in the presence of 10 mM MgSO_4_ and 0.2% maltose. Bacteriophage λ_vir_ lysate obtained on DH5α cells was added to culture aliquots at MOI of 1. After 5-min incubation, 1 μl of the mixture was placed on an agarose pad containing the M9 medium. Time-lapse filming was performed using a Nikon Eclipse Ti-E inverted microscope. Filming was carried out in transmitted light (TL) and in the mKO2 fluorescence channels. The distinct foci corresponding to phage DNA fully- or hemi-methylated at *dam* sites were visualized using the z-stacks. Each field of view was imaged every 15 min for >150 min.

### Flow cytometry

Fresh culture of *E. coli* ${\rm{DH5}}\alpha$ cells transformed with the pRM-Low plasmid was grown to OD_600_ = 0.4 in 10 ml of LB with ampicillin. 5 ml of cell culture was mixed with λ_vir_ phage lysate at the MOI = 5. Uninfected cells were used as control. Fifty-five minutes post infection cells were pelleted by centrifugation, the supernatant was discarded, and the cell pellet was resuspended in phosphate-buffered saline (PBS) to the final concentration of ∼10^6^ cells/ml.

FACS Aria III (BD) was used for fluorescence measurements. The area of bacterial cells was gated by comparing the forward and side scatter plots of PBS and plasmid-free *E. coli* DH5α cells resuspended in PBS. The 488-nm and 561-nm lasers were used for fluorescence excitation in green (Venus) and red (mCherry) channels, respectively. The Blue 530/30-A and Yel-Green 610/20-A filters were used for fluorescence detection. Selection of the region corresponding to bacterial cells was chosen by comparing the control and cell-containing samples on the side-forward scatter (SS-FS) plots. 20 000 events (fluorescence intensities of individual cells) were recorded for each sample of cells carrying the pRM-Low plasmid without and after a round of λ_vir_ infection. The obtained data were processed using Weasel and CytExpert software and the «R» programming language (http://www.R-project.org/). Samples with and without viral infection were analyzed using the Kolmogorov–Smirnov test (49). The null hypothesis was the assumption that the distributions with and without infection do not differ.

## RESULTS

### The copy number of plasmids carrying an RM system affects the level of protection from phage infection

Three plasmids carrying the Esp1396I restriction–modification system were created: pSC101_Esp1396I_fluo, pBR322_Esp1396I_fluo, and pUC19_Esp1396I_fluo. The copy numbers (PCN) for the corresponding plasmid vectors were reported to be about 5, about 20 and >100 copies per cell, respectively (50–53), and are consistent with estimates obtained using real-time PCR of total DNA samples from cells carrying Esp1396I plasmids (Supplementary Figure S1). Heretofore, we will refer to pSC101_Esp1396I_fluo, pBR322_Esp1396I_fluo and pUC19_Esp1396I_fluo as pRM-Low, pRM-Mid and pRM-High plasmids, correspondingly. The Esp1396I system cloned on each plasmid is modified by fusing the MTase and REase genes to Venus and mCherry fluorescent proteins genes, respectively. Elsewhere we show that such a modified system is functional and that both fusion proteins remain intact within the cell (36).

**Figure 1.**
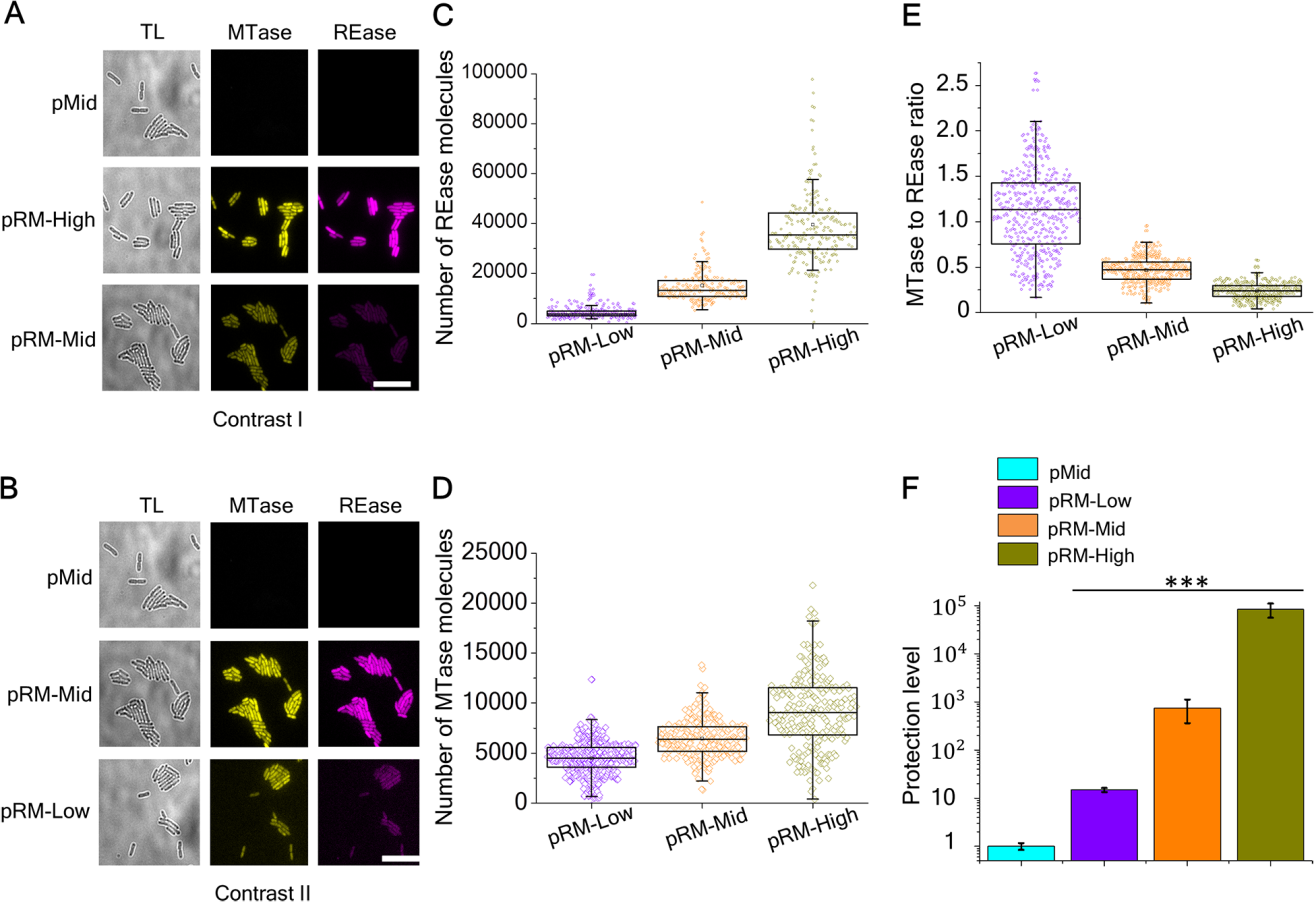
Effects of RM plasmid copy number on intracellular concentrations of Esp1396I enzymes and levels of protection from phage infection. (**A**, **B**) Images of cells with an empty pBR322 plasmid vector (‘pMid’), and cells transformed with indicated Esp1396I_fluo plasmids are shown in transmitted light (TL), Venus (yellow), and mCherry (magenta) channels. Scale bar is 20 μm. The contrasts in (**A**) and (**B**) are different to reveal the differences in fluorescence signals. (**C**–**E**) The distributions of REase (**C**) and MTase (**D**) molecule numbers and of MTase to REase ratios (**E**) in individual cells carrying the Esp1396I_fluo system on indicated plasmids. (**F**) Protection levels of cells carrying indicated RM plasmids or the pMid vector from phage λ_vir_ infection are shown. Protection levels were calculated by dividing the titer of phage lysate on the lawn of cells carrying the pMid vector by a titer determined on lawns of cells carrying an indicated plasmid. Bars represent mean protection levels obtained from three independent experiments. Error bars show standard errors of the mean. Statistical significance was assessed using Kruskal–Wallis test. ***P < 0.001.

Figure 1A and B shows microscopy images of live cells carrying fluorescently-labeled Esp1396I RM system on different plasmids in Venus and mCherry fluorescence channels. As a control, cells with an empty vector pBR322 (pMid) were used. Overall, higher fluorescence (and, therefore, higher levels of fusion proteins) were observed in cells with higher-copy number plasmids. Compared to cells carrying the pRM-High plasmid, the fluorescence intensity of cells carrying the pRM-Mid plasmid was decreased 2.6-fold in the mCherry channel and 1.4-fold in the Venus channel (Figure 1A). In cells carrying the pRM-Low plasmid, the decrease was more pronounced (9-fold and 2.1-fold, respectively) (Figure 1B).

Since in our system one molecule of Esp1396I MTase or REase corresponds to one molecule of the Venus or mCherry protein, respectively, their ratios can be determined from fluorescence intensities of individual cells. Previously, Western-blot analysis of lysates of cells carrying pRM-High with antibodies specific to Venus and mCherry proteins was carried out (36). Samples containing purified Venus and mCherry proteins of known concentration were used as standards, which allowed to estimate the average intracellular amounts of MTase::Venus and REase::mCherry fusions at ∼6000 and ∼30 000, correspondingly, and also provided coefficients for conversion of fluorescence intensities of individual cells to the number of fluorescent protein (and, therefore RM enzyme) molecules. We used this information to estimate the number of Esp1396I fusion proteins in cells containing plasmids used in this work. Our measurements of intracellular amounts of MTase::Venus and REase::mCherry fusions in cells carrying pRM-High provided average numbers of ∼8500 and 38 000, respectively, which is in good agreement with previous results (36). As can be seen from Figure 1C and D, while the absolute number of RM enzymes was proportional to PCN, as expected, the dependence of MTase and REase amounts on PCN was not the same. The correlation coefficients between REase::mCherry and MTase::Venus amounts in individual cells were 0.48 (*n* = 666) in cells with pRM-High, 0.45 (*n* = 690) in cells with pRM-Mid, and 0.37 (*n* = 721) in cells with the pRM-Low plasmid. As a result, the mean MTase to REase ratio values increased with decreasing PCN (Figure 1E). The widths of MTase to REase ratio distributions in individual cells were wider in cells with the lower copy number plasmids and the effect was most pronounced for cells carrying pRM-Low (Figure 1E). While the concentration of the Esp1396I C protein that orchestrates the complex regulation of transcription of Esp1396I MTase and REase genes was not measured, it is likely that the observed effects are caused by lower C protein concentrations in cells carrying lower copy number plasmids, which should lead to higher relative MTase expression levels and larger stochastic effects (30).

Protection levels of cells carrying different RM plasmids or empty vector controls were tested by applying drops of serial dilutions of phage λ_vir_ (harbors 14 Esp1396I recognition sites of which only one is protected by overlapping Dcm methylation) lysate on the surfaces of lawns formed by different cells. The level of protection was calculated as a ratio of phage titers determined on lawns of cells with RM plasmids to the titer observed on control cell lawns. As expected, no difference in resistance was observed between control cells carrying different empty vectors (Supplementary Figure S2). As can be seen from Figure 1F, bacteria harboring the pRM-Low plasmid were least protected (∼10-fold decrease in phage titer), while bacteria carrying the pRM-High plasmid were most protected (∼5 × 10^4^-fold phage titer decrease). Cells carrying the pRM-Mid plasmid had an intermediate, almost 10^3^-fold protection level. We conclude that there is a positive correlation between the copy number of an Esp1396I RM system-encoding plasmid and the protection level from phage infection. Similar results were obtained by others with a different Type II RM system (54). Despite the low protection level of cultures carrying the pRM-Low plasmid (Figure 1C), the progeny phage formed upon a single-round of infection of these cells productively infected the highly protected pRM-High cultures and hence was modified (Supplementary Figure S3).

### The influence of additional REase or MTase enzymes on protection from bacteriophage infection

**Figure 2.**
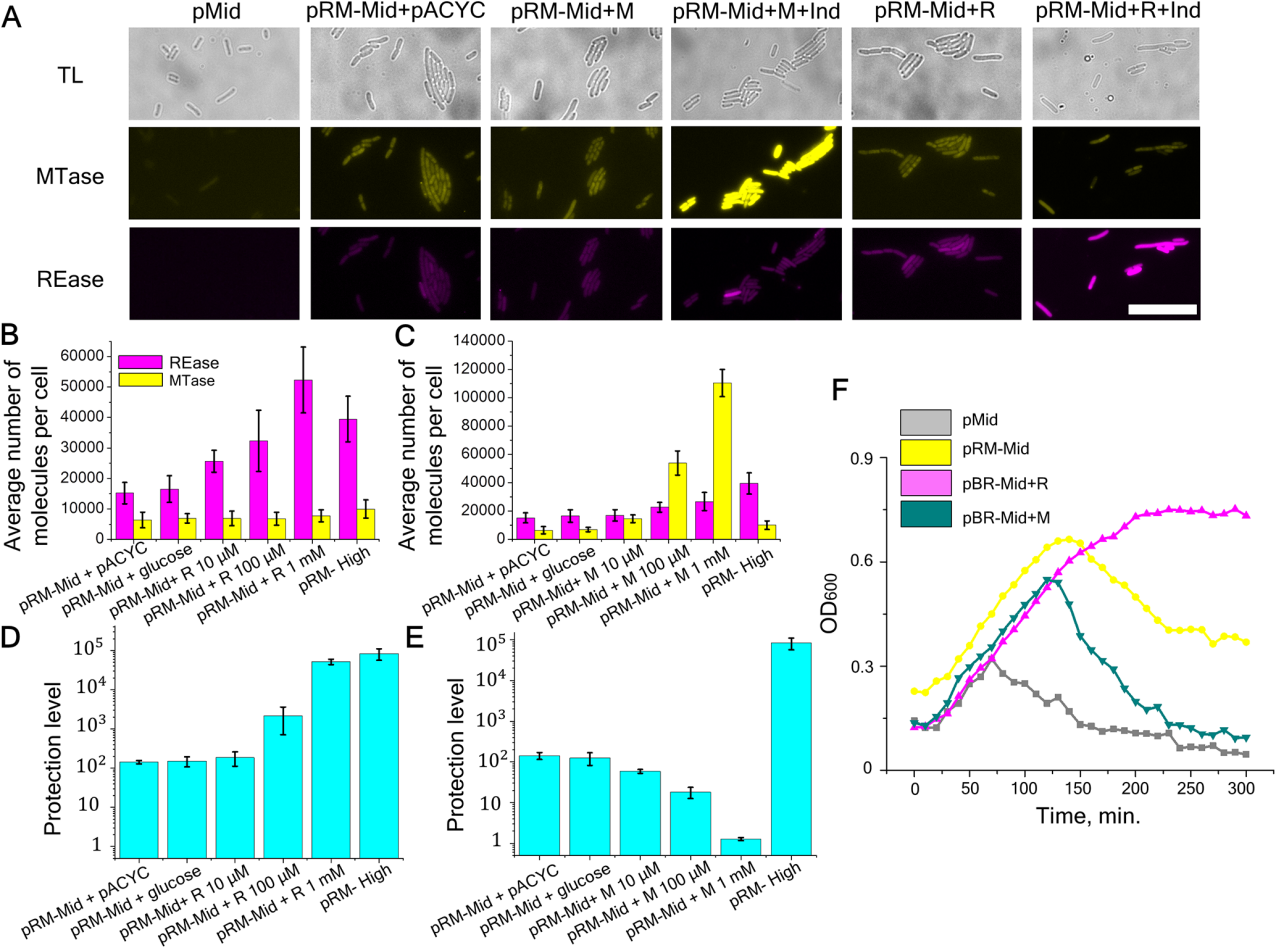
The influence of additionally induced Esp1396I enzymes on the level of protection. (**A**) Images of cells in transmitted light (gray) and in two fluorescence channels Venus (yellow) and mCherry (magenta): pMid - control cells without the RM system; pRM-Mid+ pACYC – cells with pRM-Mid and pACYC plasmids; pRM-Mid+ M – cells with pRM-Mid and +M plasmids with or without 10 mM arabinose induction; pRM-Mid+ R – cells with pRM-Mid and +R plasmids with or without 10 mM arabinose induction). Scale bar is 20 μm. (**B, C**) Intensities of fluorescence in 300 individual cells with additional synthesis of REase (**B**) or MTase (**C**) induced by different concentrations of arabinose. Yellow and magenta bars represent the mean number of MTase and REase molecules per cell, respectively. Error bars represent standard error of the mean. (**D, E**) Protection from phage infection of cells with additionally expressed REase (D) or MTase (**E**). Protection levels were determined as described in Figure 1F legend. Error bars represent standard error of the mean. (**F**) Growth of cultures of cells without RM protection (pMid), cells carrying the pRM-Mid plasmid, and pRM-Mid carrying cells expressing additional MTase (pRM-Mid + M) or REase (pRM-Mid + R). Cells were grown in the presence of 1 mM arabinose. At zero time point, cultures were infected with λ_vir_ phage at a MOI of 0.2 (the arrow indicates start of infection).

Our results show that the higher the copy number of a plasmid carrying the Esp1396I system, the higher is the average concentration of both RM enzymes in the cells and the level of protection against viral infection. However, when the copy number of a plasmid carrying the RM system is increased, the relative concentration of REase increases to a higher extent than that of the MTase, resulting in a decrease of the MTase to REase ratio (Figure 1D–F). To find out how changes in the concentration of only one RM enzyme would affect protection against the virus, gene fusions encoding the REase::mCherry or the MTase::Venus proteins were placed downstream of the arabinose-inducible promoter on pACYC plasmids compatible with the pRM-Mid plasmid. We will refer to the resulting pACYC_R_fluo and pACYC_M_fluo plasmids as ‘+R’ and ‘+M’, respectively. Bacteria co-transformed with pRM-Mid and either +R or +M plasmids were analyzed by fluorescence microscopy in the presence or in the absence of a 10 mM arabinose inducer. As a control, cells without the RM system plasmid and cells that carried the pRM-Mid plasmid and the empty pACYC vector were used. As can be seen from Figure 2A, induction of the MTase gene expression led to increased fluorescence in the Venus channel compared to control bacteria containing the pRM-Mid plasmid and the empty vector, as expected. Also, as expected, an increase in fluorescence in the mCherry channel was observed upon induction of additional REase synthesis.

The increase in REase or MTase production in cells carrying additional expression plasmids as a function of arabinose inducer concentration was measured 2 h post-induction using fluorescence microscopy. As controls, we used (i) cells carrying empty pMid and pACYC vectors; (ii) cells with the pRM-Mid plasmid and the pACYC vector and (iii) cells with the pRM-Mid plasmid and inducible REase or MTase expression plasmids grown in the presence of 0.2% glucose, a condition that should completely repress transcription from the arabinose promoter. Mean fluorescence values for 300 cells from each sample were determined and results are presented in Figure 2B. For cells harboring the inducible REase fusion, induction with 0.01 and 0.1 mM arabinose led to, respectively, 1.5- and 1.7-fold increase of mean fluorescence intensity in the mCherry channel compared to cells with the pRM-Mid plasmid and the pACYC vector or cells grown in the presence of glucose. A 2.7-fold increase was detected in cells induced with 1 mM arabinose. Further increase of inducer concentration did not lead to additional increase in mean fluorescence intensity (data not shown). No increase in mean fluorescence in the Venus channel in samples with additionally induced REase was observed, indicating that levels of MTase remain constant. In cultures of cells with inducible MTase, mean fluorescence intensity in the Venus channel in the presence of 0.01, 0.1 and 1 mM of arabinose increased 1.8-, 8.7- and 18-fold, respectively (Figure 2C). In the presence of 0.1 and 1 mM arabinose, a moderate (1.6–1.7-fold) increase in the level of REase was also detected. Overall, we conclude that the use of additional plasmids expressing REase or MTase allows us to alter the ratio of RM enzymes without significantly affecting the concentration of the counterpart protein expressed from the RM system-carrying plasmid. The concentration of REase in cells harboring the pRM-Mid plasmid and expressing additional REase in the presence of 0.1–1 mM arabinose approaches that in cells harboring the pRM-High plasmid (Figure 2B). The concentration of MTase in cells harboring the pRM-Mid plasmid and expressing additional MTase at 1 mM arabinose is several-fold higher than that in cells harboring the pRM-High plasmid (Figure 2C).

We next determined the level of protection from phage infection of cells with different MTase to REase ratios. In samples with inducible REase, the presence of 0.01, 0.1 and 1 mM arabinose increased the level of protection by 0.6, 1 and 2 orders of magnitude, respectively, compared to that of cells carrying the pRM-Mid plasmid and the pACYC vector (Figure 2D). At 1 mM arabinose, the protection was equal to that observed in cells harboring the pRM-High plasmid. The increase of inducer concentration to 10 mM did not lead to additional protection (data not shown). Conversely, induction of additional MTase synthesis decreased the protection, which disappeared completely at or above 1 mM arabinose (Figure 2E).

We also monitored the growth of cells carrying the RM system on the pMid plasmid, with or without additional REase or MTase expression plasmids, and control cells carrying empty vectors upon infection with phage λ_vir_ (multiplicity of infection (MOI) = 0.2) (Figure 2F). Cells were induced with 1 mM arabinose 2 h prior to infection. As can be seen, the unprotected culture started to lyse ∼70 min post-infection, presumably after the second cycle of phage infection. Cells carrying the RM system plasmid and the pACYC vector continued to grow for ∼150 min and then started to lyse, presumably due to accumulation of the modified phage. Induction of additional REase made the culture fully resistant to the phage. Induction of extra MTase synthesis made cells more sensitive to infection compared to cells with the pRM-Mid plasmid as the only source of RM enzymes: lysis started 120 min post-infection and the culture lysed completely by 300 min post-infection when the culture with the pRM-Mid plasmid as the only source of RM enzymes was only partially lysed. Together, these results show that the decrease in the Esp1396I MTase to REase ratio increases the level of protection from phage infection. Conversely, the increase in MTase to REase ratio decreases the level of protection.

### The influence of PCN and additional expression of REase or MTase on bacteriophage λ DNA degradation after infection of cells carrying the Esp1396I_fluo RM system

To determine the mechanism of changes in protection levels in cells carrying the Esp1396I_fluo system on different copy number plasmids and with additionally expressed individual RM enzymes, we followed the fate of injected phage DNA inside infected cell. To this end, we used the ability of the SeqA protein to specifically bind to Dam methylated or hemimethylated DNA. The K-12 MG1655 host strain used for this experiment was *dam-* and constitutively expressed SeqA fused with the mKO2 fluorescent protein (SeqA::mKO2). The DNA of the phage used for infection was fully Dam methylated. Since the MG1655 host is *recA+*, while the DH5α cells used in previous experiments are *recA-*, we first determined the protection levels of K-12 MG1655 cells carrying pRM-High, pRM-Mid and pRM-Low from infection with λ_vir_. As can be seen from Supplementary Figure S4, similarly to the result with DH5α, protection levels of K-12 MG1655 cells increased with the RM plasmids PCN. The actual levels of protection of K-12 MG1655 were several-fold less than that of DH5α carrying a comparable plasmid, with the effect most pronounced for pRM-Low. Since both strains carry high and similar number of Esp1396I recognitions sites in their genomes (1644 in DH5α and 1657 in MG1655) and, when transformed with pRM-Mid, grow at similar rates at our conditions (Supplementary Figure S5), the effect is likely due to either direct influence of RecA-dependent processes on repair of DNA cleaved by Esp1396I or on plasmid copy numbers (or both). Indeed, plasmid copy numbers in K-12 MG1655 were decreased compared to those in DH5α (compare Supplementary Figure S4B and S1). Be that is it may, we conclude that data obtained with the two different strains can be qualitatively compared with each other.

**Figure 3.**
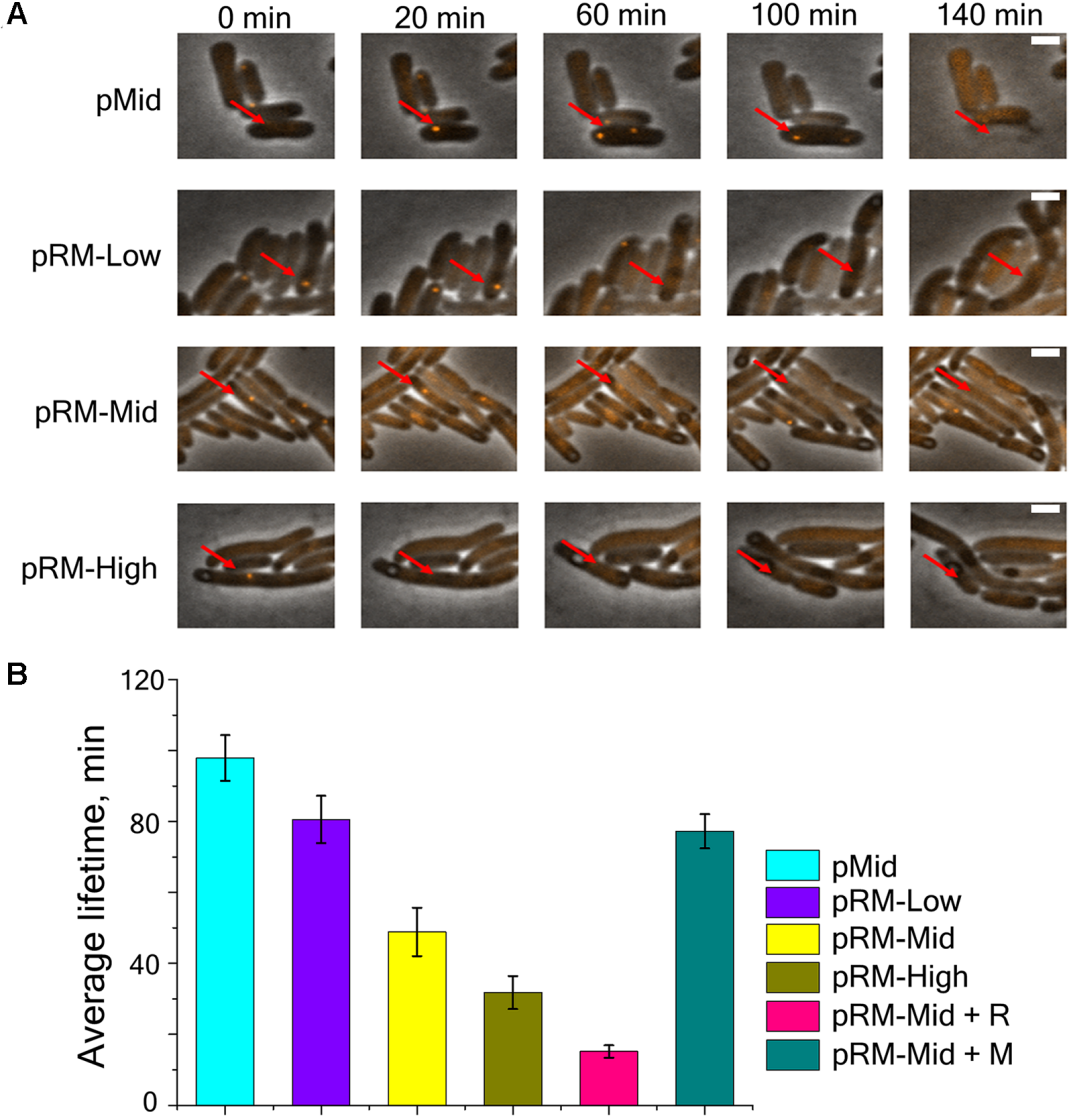
Degradation of phage DNA in single cells protected by Esp1396I. (**A**) Images from a time-lapse movie showing the appearance of SeqA::mKO2 foci in cells from indicated cultures. Red arrow points to cells infected with phage. Scale bar is 2 μm. (**B**) Average foci lifetimes in infected cells carrying indicated plasmids. Mean values obtained upon observation of 690 infected cells carrying empty pMid plasmid, 43 cells carrying the pRM-High plasmid, 128 cells carrying the pRM-Mid plasmid, 133 cells carrying the pRM-Low plasmid, 214 induced cells carrying pRM-Mid and +M plasmids, and 49 induced cells carrying the pRM-Mid and +R plasmids are shown. Error bars represent standard errors of the mean.

Cells carrying pRM-Low, pRM-Mid or pRM-High plasmids or cells carrying the pRM-Mid plasmid with either +R or +M plasmids supplemented with 1 mM arabinose as well as appropriate control cells were infected with bacteriophage λ_vir_ at the MOI = 1. As is described elsewhere (48), SeqA::mKO2 binds to fully methylated injected phage DNA forming a distinct focus visible in the mKO2 channel (Figure 3A). When phage DNA is replicated, the number of foci increases to two per each injected phage genome and remains constant throughout the infection until the cell lyses. Accordingly, cells were imaged in transmitted light and in the mKO2 fluorescent channel every 10 min after the infection.

In pMid cells and in induced pRM-Mid +M cells, the appearance of a single mKO2 focus shortly after the beginning of observation was detected followed by the appearance of the second focus, indicating replication of injected phage DNA. Such cells eventually lysed (Figure 3A). For unprotected pMid cells, an average focus lifetime was 97 min (Figure 3B, foci lifetimes were calculated from the first moment of appearance of a focus until cell lysis). In cells harboring RM plasmids (with the exception of pRM-Mid +M cells), only single foci were observed. Bacteria with these foci did not lyse; instead the foci eventually disappeared (Figure 3A). The average foci lifetimes (measured from the time of focus appearance till its disappearance) in cells harboring pRM-Low, pRM-Mid and pRM-High plasmids were 80, 49 or 31 min, respectively. For pRM-Mid samples with additionally induced REase, this value was 18 min (Figure 3B). For pRM-Mid samples with additionally induced MTase, the focus lifetime (determined by the time of cells lysis) was 82 min (Figure 3B).

### Determination of REase and MTase levels in productively infected bacteria

**Figure 4.**
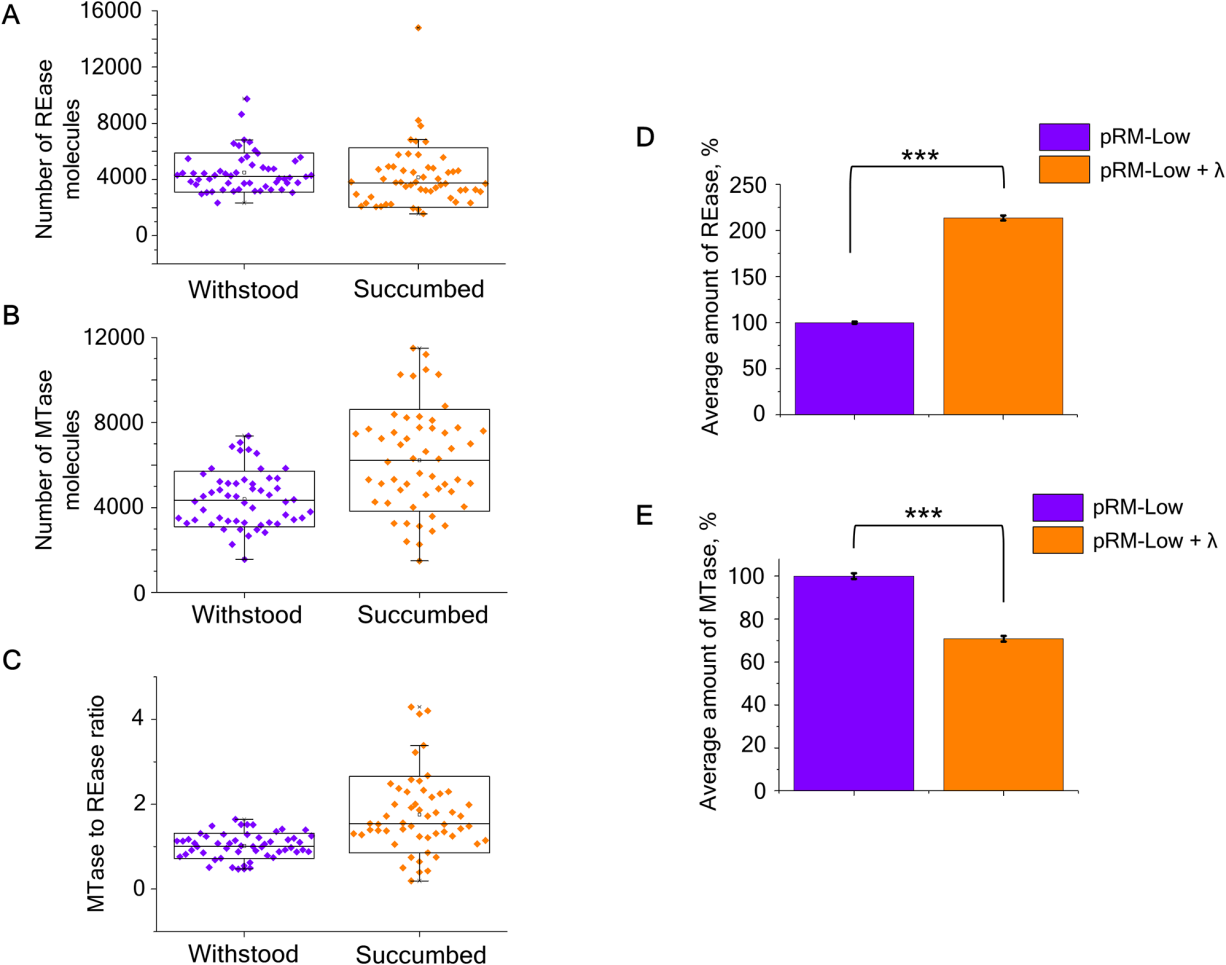
Distribution of enzymes in cells with the pRM-Low plasmid that either succumbed to or withstood phage infection. Distribution of REase (**A**) and MTase (**B**) molecule numbers and of MTase to REase ratios (**C**) in individual productively infected (orange box charts) and withstood (violet box charts) cells carrying the Esp1396I_fluo system on indicated plasmids at the time of the infection. (**D**) Average amounts of REase in cells carrying the pRM-Low plasmid without (purple) and after (orange) a round of λ_vir_ infection measured by flow cytometry. Average fluorescence in the red fluorescence channel of non-infected cells transformed with the pRM-Low plasmid was taken as 100% of the fluorescent signal. (**E**) Average amounts of MTase in cells carrying the pRM-Low plasmid without (purple) and after (orange) a round of λ_vir_ infection measured by flow cytometry. Average fluorescence in the green fluorescence channel of non-infected cells transformed with pRM-Low plasmid was taken as 100% of the fluorescent signal.

We next determined whether there is a specific subpopulation of cells in RM-protected cultures that is more efficiently attacked by unmodified phage. Based on results presented above, cells in such a subpopulation, if it exists, should have a higher MTase to REase ratio than cells in the rest of the population. We infected bacteria transformed with the pRM-Low plasmid with bacteriophage λ_vir_ and followed the infection using live fluorescence microscopy. The pRM-Low plasmid was used because protection levels afforded by pRM-Mid and pRM-High plasmids were too high, making it impossible to observe enough rare productive infection events under the microscope. Infection was carried out at the MOI = 1. While 33% of cells transformed with the pLow plasmid were lysed after a round of infection, only 7% of cells carrying the pRM-Low plasmid lysed, in agreement with the protection levels determined using the drop test method (Figure 1C). Given that the experiment was conducted at high MOI, we surmise that most surviving RM-bearing cells were infected at the beginning of the experiment but managed to destroy injected phage DNA. We estimated the overall levels and the ratios of MTase and REase fusions in 54 RM-carrying cells that succumbed to the infection and in the same number of cells that withstood it (Figure 4A–C). the REase and MTase amounts were measured at the first frame, taken 10 min after infection.

The mean numbers of REase fusion molecules in productively infected cells and cells that withstood the infection were, respectively, 4155 ± 288 and 4500 ± 190 per cell, which is statistically indistinguishable. However, the difference in mean numbers of MTase fusion molecules in infected cells and cells that withstood the infection (6219 ± 326 and 4400 ± 178, respectively) was highly significant (*P* < 0.001) and led to a shift of the MTase to REase ratio in productively infected cells to higher values compared to cells that withstood the infection (1.75 ± 0.123 versus 1.02 ± 0.04).

As an alternative method to investigate whether a round of phage infection changes the MTase to REase ratio distribution in a surviving bacterial population, infected and uninfected cultures of cells carrying the pRM-Low plasmid were analyzed by flow cytometry. Infection was performed at a high MOI = 5 such that all cells were expected to be infected at the start of the experiment. A shift in the distribution of fluorescence intensities in cells upon the infection was detected. First, a significant increase (two-sample Kolmogorov–Smirnov test *P*-value < 2.2 × 10^–16^) in mean fluorescence intensity in the red fluorescence channel corresponding to REase was observed (Figure 4D). Second, there was a significant (Kolmogorov–Smirnov test *P*-value < 2.2 × 10^–16^) decrease (Figure 4E) in mean fluorescence intensity in the green fluorescence channel corresponding to MTase. We conclude that cells with stochastically higher concentrations of REase or lower concentrations of MTase become more prevalent in the population of cells with the plasmid-borne RM system after one round of infection with the phage. This observation supports a view that cells with a larger than average amount of MTase and a lower-than-average amount of REase are more likely to be infected by the phage.

## DISCUSSION

In this work, we demonstrate that changing the ratio of Type II Esp1396I RM system MTase and REase from the one afforded by its natural homeostatic transcription regulation mechanism (30,35) can either completely abolish protection from viral infection (when the amount of Esp1396I MTase is increased) or make all cells resistant to the infection (when the amount of Esp1396I REase is increased). The mechanistic explanation for these observations is provided by direct measurements of half-lives of injected unmodified phage DNA, that show that production of additional Esp1396I REase reduces the average time needed for degradation of phage DNA thus enhancing the level of protection. On the contrary, in cells producing additional MTase, the average time needed for invading phage DNA degradation is increased, ultimately abolishing protection. Likewise, increasing the copy number of plasmids carrying the Esp1396I RM system increases the protection of cells from phage infection and also leads to faster degradation of injected phage DNA.

Our results confirm the intuitive expectations and show that the balance of MTase and REase activity is critical for protection from genetic predators. Similar trends were observed during analysis of λ phage infection of cells carrying plasmid-borne EcoVIII system, when plasmid copy number was affected by mutations in the plasmid or host bacterium genes (54). The magnitude of effects was smaller in the EcoVIII study, which may be related to the different number of recognition sites in the λ phage genome (6 for EcoVIII versus 14 for Esp1396I) or different modes of regulation of the EcoVIII enzyme production, which occurs not through C protein control but through the presence of rare arginine codons that limit the amount of EcoVIII enzymes produced (55). Both factors could lower the effective activity of EcoVIII enzymes thus affecting the overall protection levels.

Wilkowska *et al.* reported that for the EcoRI system, higher copy number of plasmids carrying the RM system genes actually led to decreased protection from bacteriophage infection (20). However, this apparently paradoxical behavior was due to the fact that cells carrying higher PCN plasmids with the EcoRI system produced less REase than cells with lower copy number plasmids (20). Moreover, cells with higher, but not lower copy number EcoRI plasmids underwent SOS response, suggesting that their DNA was undermethylated and attacked by the REase. Indeed, expression of additional EcoRI MTase from a separate plasmid decreased the SOS response and also abolished the residual level of protection from infection. The latter result is in full agreement with our data. The amounts of EcoRI MTase were unfortunately not determined in the work of Wilkowska *et al.*, making direct comparison with our data not possible. Presumably, the difference between the Wilkowska *et al.* and our data is explained by the complex regulation of *ecoRI* genes expression, which occurs through multiple promoters, including intragenic promoters, which encode antisense transcripts that target the sense transcripts of the *ecoRI* genes (56,57). Apparently, changes in plasmid PCN affect expression of the *ecoRI* genes in complex ways that remain to be determined.

Similar effects, albeit of a much smaller magnitude, may be operational in other C protein-dependent system studied. While C proteins are most important during establishment of an RM system in a naïve host, ensuring that no REase is produced before host DNA is modified by the MTase (36,58), they can also control the amounts of REase and MTase at steady-state, when a plasmid carrying the RM system is established. We speculate that changes in Esp1396I C protein concentration upon the change in PCN (i.e. gene copy number) are responsible for observed changes in MTase/REase ratios as a function of PCN. Indeed, inducible expression of Esp1396I C protein from a separate plasmid decreased protection of cells carrying pRM-Mid from phage infection by as much as 10-fold ((59) and our unpublished observations). To further understand these effects, one would need to monitor the intracellular amounts of Esp1396I C protein and we are currently developing such a system. Be it as it may, our data and the data of others suggest that it shall be highly informative to determine the effects of PCN on the protective behavior of RM systems with different mechanisms of regulation.

RM system-specific differences noted above notwithstanding, analysis of changes in protection levels at different induction levels of MTase and REase genes shows that the MTase to REase ratios strongly affect the level of protection. It is interesting to compare the observed effects of variation in experimentally determined intracellular REase and MTase amounts on phage infection with quantitative theoretical predictions made in (34). We note that the theoretical analysis considered enzymatic activities of RM enzymes, while in our work the amounts of REase and MTase inside the cells were determined. Yet, the ratio of the amounts of the two enzymes is related to their activity ratios up to a constant factor. While this constant is unknown and may be affected by different parameters, including the stoichiometry of active REase and MTase, which in the case of Esp1396I is unknown, it shall be possible to determine how well the equation described in (34) and relating phage survival probability to REase and MTase ratio fits experimental data. The equation

(1) $$\begin{equation*}P = {\left( {\frac{\mu }{{\mu + \rho }}} \right)^N} = {\left( {1 + \tau \frac{{{N_{REase}}}}{{{N_{MTase}}}}} \right)^{ - N}}\end{equation*}$$

expresses the phage survival probability P through the probabilities of methylation μ and restriction ρ of all N restriction-methylation sites present in the phage genome. The probabilistic meaning of Equation (1) is quite transparent: a phage survives and produces (modified) progeny if and only if each of the N sites becomes methylated and no site is cleaved by a REase. The cellular environment is assumed to be uncrowded so that enzymatic reactions at a given recognition site are independent of the fate of other sites. It is further assumed that μ and ρ are proportional to the corresponding numbers of MTase and REase molecules within the cell, i.e. $\mu = {\alpha _{MTase}}{N_{MTase}}$ and $\rho = {\alpha _{REase}}{N_{REase}}$_,_ with factor τ defined as the ratio of proportionality coefficients, $\tau = {\alpha _{REase}}/{\alpha _{MTase}}$.

**Figure 5.**
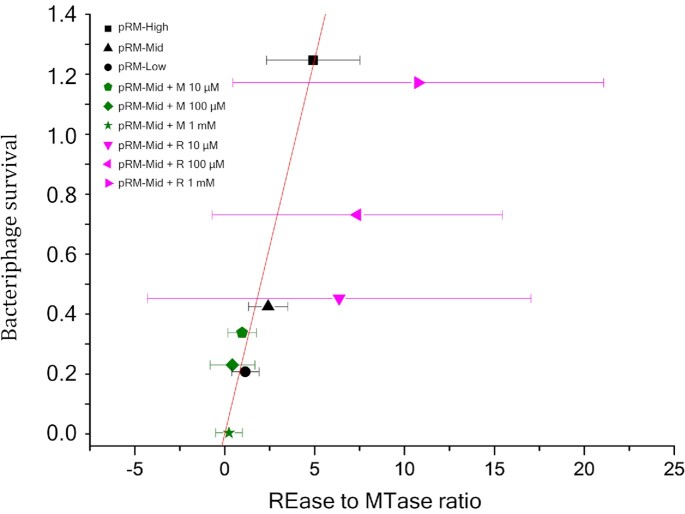
Bacteriophage survival probability. The least square linear fit *y* = *τx* to three datasets shown in black (bacterial cells with pRM-High, pRM-Mid, pRM-Low plasmids), green (pRM-Mid + M– cells with pRM-Mid and +M plasmids in presence of different concentrations of arabinose), and magenta (pRM-Mid + R– cells with pRM-Mid and +R plasmids in presence of different concentrations of arabinose). The fit has the slope τ = 0.25 and is shown by a red line. For each dataset *N* = 14 and the respective average and standard deviation are shown by a symbol and horizontal error bars.

To see how well Equation (1) describes phage survival probability observed in experiments with cells harboring the Esp1396I_fluo system on plasmids with different PCN or cells expressing additional MTase or REase, we plotted

(2) $$\begin{equation*}{P^{ - 1/N}} - 1\,{\rm{vs}}.\frac{{{N_{REase}}}}{{{N_{MTase}}}}\end{equation*}$$

for three data sets: (i) data obtained with cells harboring the RM system on high, middle or low copy number plasmids; ii) data obtained with cells carrying the RM system encoded on a pRM-Mid plasmid with additional expression of MTase from the +M plasmid and grown at different concentrations of arabinose inducer, and iii) data obtained with cells carrying the pRM-Mid plasmid and the +R plasmid grown at different concentrations of inducer. The results are presented in Figure 5. As can be seen, a fit *y* = *τx*, *τ* = 0.25, expected to be linear from Equation (1), is very good and passes well within the standard deviations of each data set. This indicates that a simple Equation (1) indeed captures the essence of REase and MTase kinetics and predicts phage survival in cells harboring plasmid-borne Esp1396I RM system. It should be noted that the statistical quality of the pRM-Mid + R dataset (magenta) noticeably differs from that of two other data sets (black and green) (Figure 5) and a significant increase in standard deviation is observed in addition to the expected increase in the REase to MTase ratio values. Microscopic analysis of individual pRM-Mid + R DH5α cells indicated that ∼7% of cells contained very high levels of REase and approximately one third of these very bright cells became elongated (Supplementary Figure S6A). Yet induction of additional synthesis of Esp1396I REase has no effect on cell growth (Supplementary Figure S6B). While the exact cause of the apparent dysregulation of REase synthesis in the minority of pRM-Mid +R cells was not investigated, it certainly contributed to the increase of standard deviation since all cells were included in the analysis.

Available data show that actual levels of protection of cells with RM systems vary depending on the system and its location (chromosome versus plasmid) and must be subject to evolutionary pressures. While the exact nature and extent of these pressures remains to be defined, the increased amounts of REase needed to achieve complete protection from phage infection likely decrease the viability of cells by allowing occasional attack on host own DNA whenever unmethylated sites appear. Consequences of MTase/REase imbalance were studied mostly by following phenotypes of bacteria harboring Type II RM systems carried on different copy number plasmids (20,54) or overproducing REase (60,61) or MTase (35). In general, the high expression level of RM enzymes led to SOS response and cell death, mostly due to the star activity of REases which was not contained by methylation (19,20,60–62). The activity levels of RM enzymes may be modulated in complicated ways, for example by different localization of MTase and REase within the cell. For C protein-dependent RM system Csp231I it was shown that while REase molecules freely diffuse in the cytoplasm, the MTase molecules are mostly located near the nucleoid (58). With Esp1396I, the situation appears to be similar (36). The difference in localization may increase the efficiency of target location by MTase, allowing to protect host sites from REase action with relatively small amount of MTase molecules. This should help prevent unwanted methylation of incoming viral DNA and also allow to increase the amount of REase able to provide efficient defense.

Our demonstration that alterations in the MTase to REase ratio can drastically affect the level of phage protection provides an explanation of how the protection afforded by RM systems can be overcome in clonal cell populations. Stochastic variation in intracellular amounts of RM enzymes leads to increased susceptibility of individual cells to infection, and DNA of progeny phage of such infection events are modified, allowing the virus to take over the initially protected population of cells. Indeed, our real-time analysis of individual infected cells harboring the Esp1396I system on a low copy number plasmid shows that productively infected cells at the time of infection have relatively more MTase than cells that ward off the infection. Thus, the tight regulation of Type II RM systems genes transcription serves not just to establish a correct temporal sequence of MTase (first) and REase (second) synthesis during the establishment of an RM system-carrying plasmid in a naïve host (36), but also affords the highest possible level of protection by allowing stable and robust levels of expression of both genes (55,58,63,64).

## DATA AVAILABILITY

Materials and reagents described in this article are freely available from the authors upon request. Datasets from Flow Cytometry experiments have been deposited in FlowRepository (Repository ID: FR-FCM-Z55J, http://flowrepository.org/id/FR-FCM-Z55J).

## Supplementary Material

gkac1124_Supplemental_File
