# Supplementary material for: Cells with stochastically increased methyltransferase to restriction endonuclease ratio provide an entry for bacteriophage into protected cell population
Source: Nucleic Acids Res. 2022 Dec 8;50(21):12355–68. doi: 10.1093/nar/gkac1124 (PMC9757035; doi:10.1093/nar/gkac1124)
Supplement: gkac1124_Supplemental_File [file gkac1124_supplemental_file.docx]

**Supplementary material**


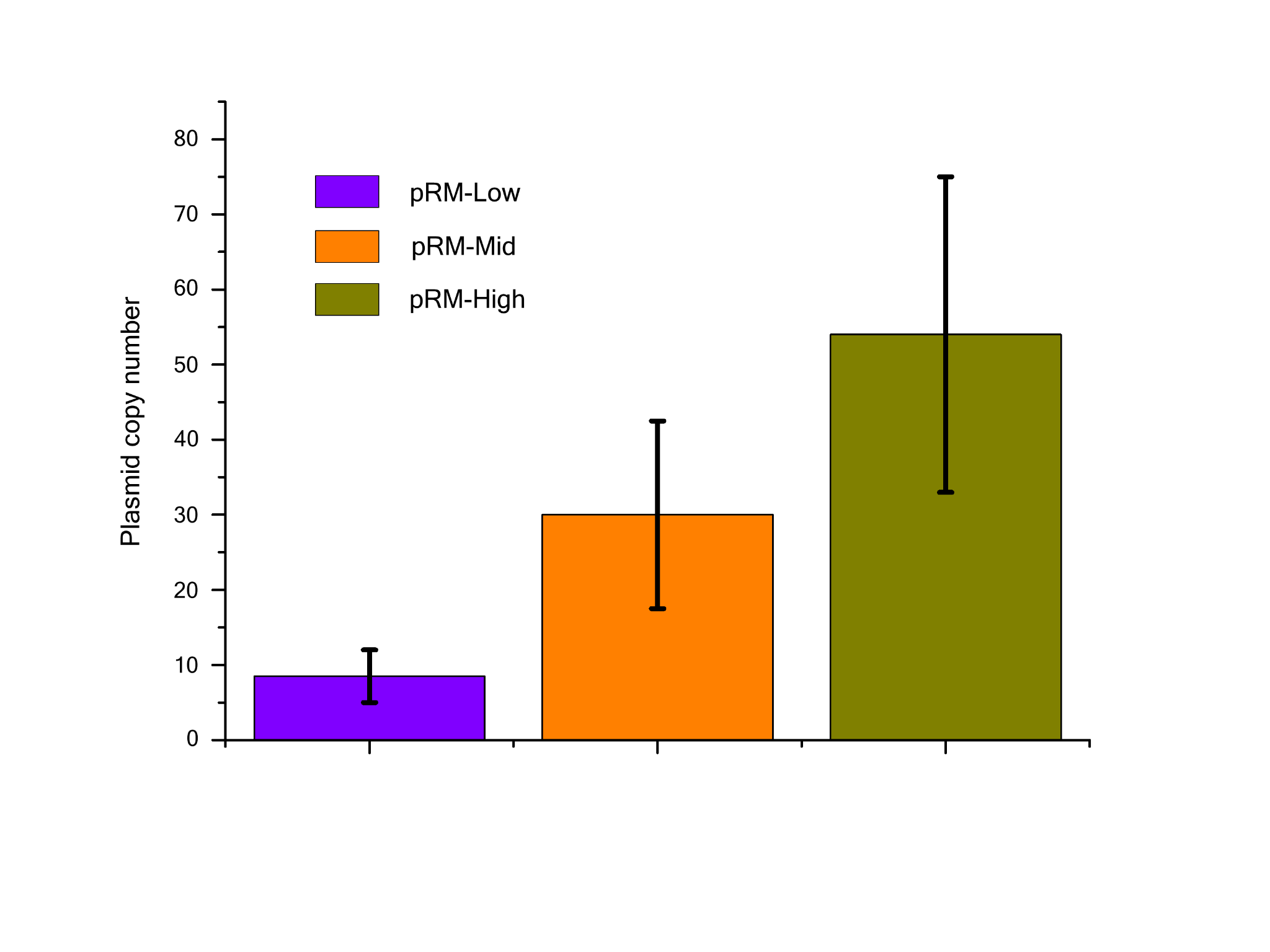
**Supplementary Figure S1**. The copy number of pRM-Low, pRM-Mid, and pRM-High plasmids in *E. coli* DH5𝛼 cells.

The plasmid copy number (PCN) was determined by quantitative real-time PCR (qPCR) using the ΔC_t_-method (1). The concentrations of plasmid and chromosomal (*E. coli* DH5𝛼) DNA were determined using two separate primer sets, one specific for plasmid-borne Esp1396I methyltransferase gene and another - for the *gyrA* gene (coding for the DNA gyrase subunit) (primer sequences are provided in Supplementary Table S1). To calculate the absolute concentration of plasmid and genomic amplicons, efficiency of PCR was estimated from standard curves obtained from qPCR reactions with aliquots of serial dilutions of solutions containing known concentrations of purified pRM-High or chromosomal DNA as templates. PCN was calculated by taking a ratio of absolute concentrations of plasmid and genomic DNA in the sample. Error bars represent standard deviations and the bars show mean values obtained from measurements performed for three independent cultures.


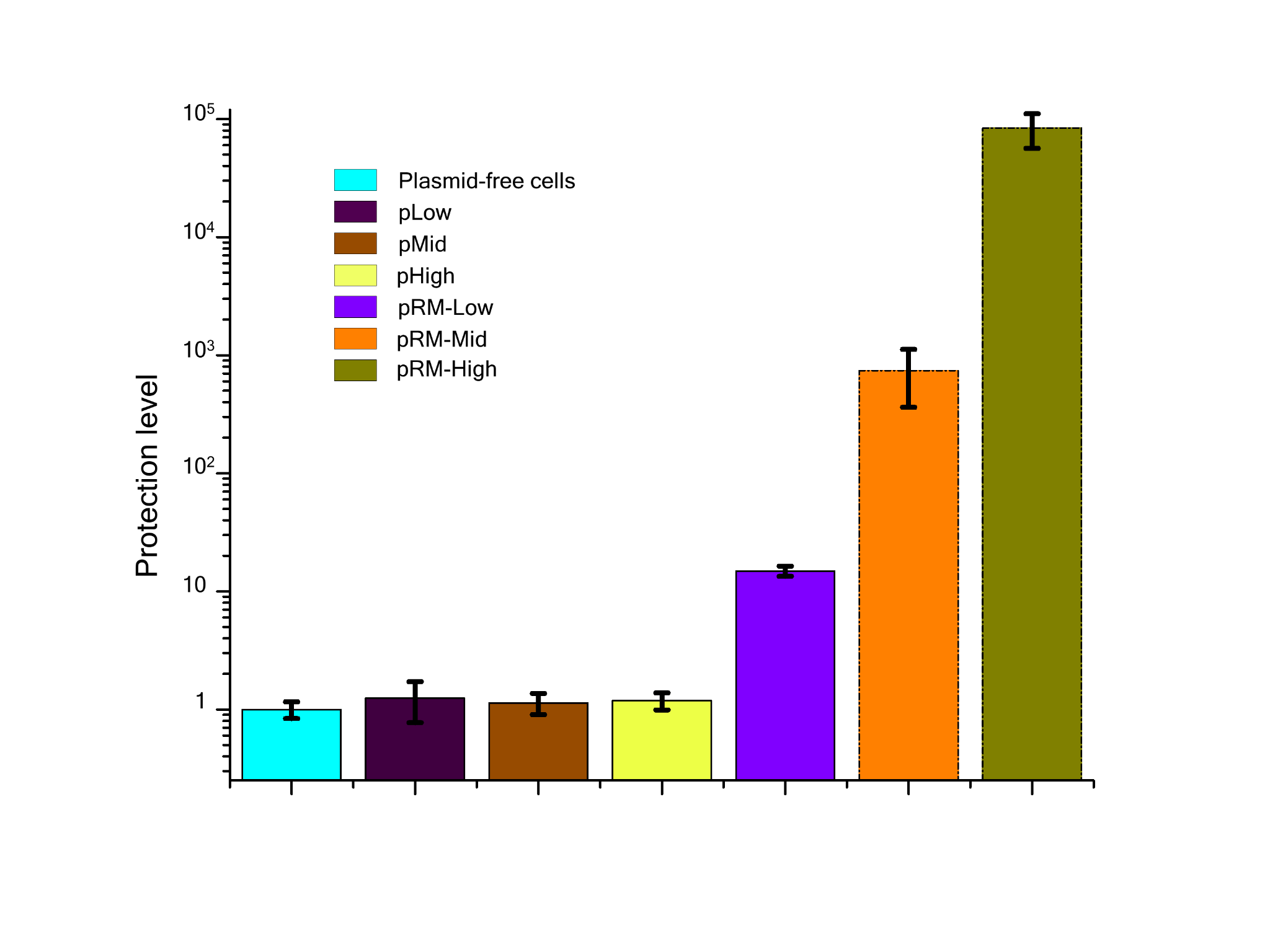


**Supplementary Figure S2.** Protection of plasmid-free DH5𝛼 *E. coli* cells and cells carrying indicated plasmids from phage λ_vir_ infection.

Protection levels were calculated by dividing the titer of phage lysate on the lawn of plasmid-free DH5𝛼 cells by a titer determined on lawns of cells carrying indicated plasmids. Bars represent mean protection levels obtained from three independent experiments. Error bars show standard errors of the mean.

**
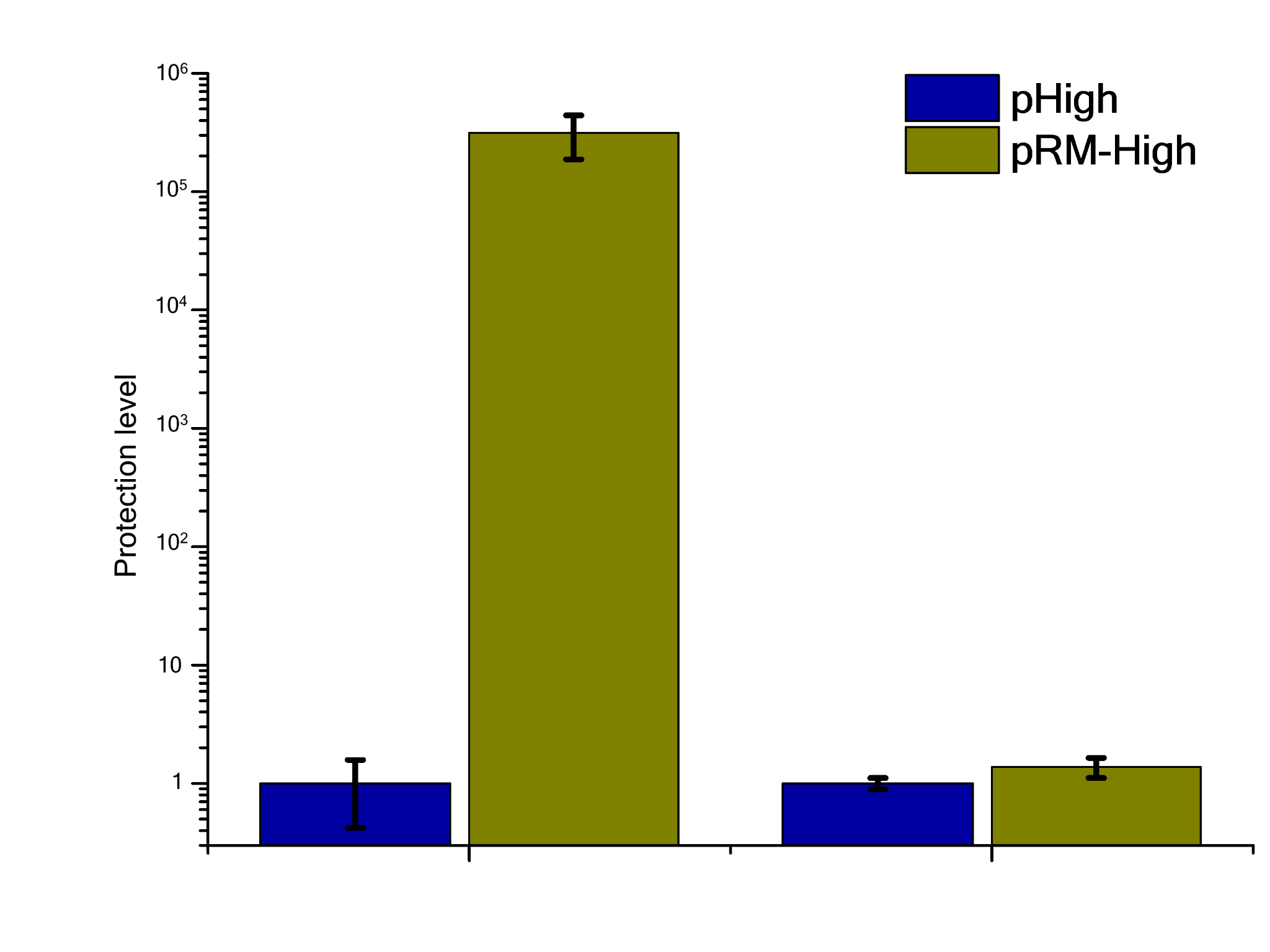
Supplementary Figure S3.** Protection level of cells carrying the pHigh vector control (blue-coloured bars) or the pRM-High plasmid (olive-coloured bars) from infection by the λ_vir_ phage obtained after a single round of infection of cells without an RM system (two columns on the left) or cells carrying the pRM-Low plasmid (two columns on the right). Bars represent mean protection levels obtained from three independent experiments. Error bars represent standard errors of the mean.


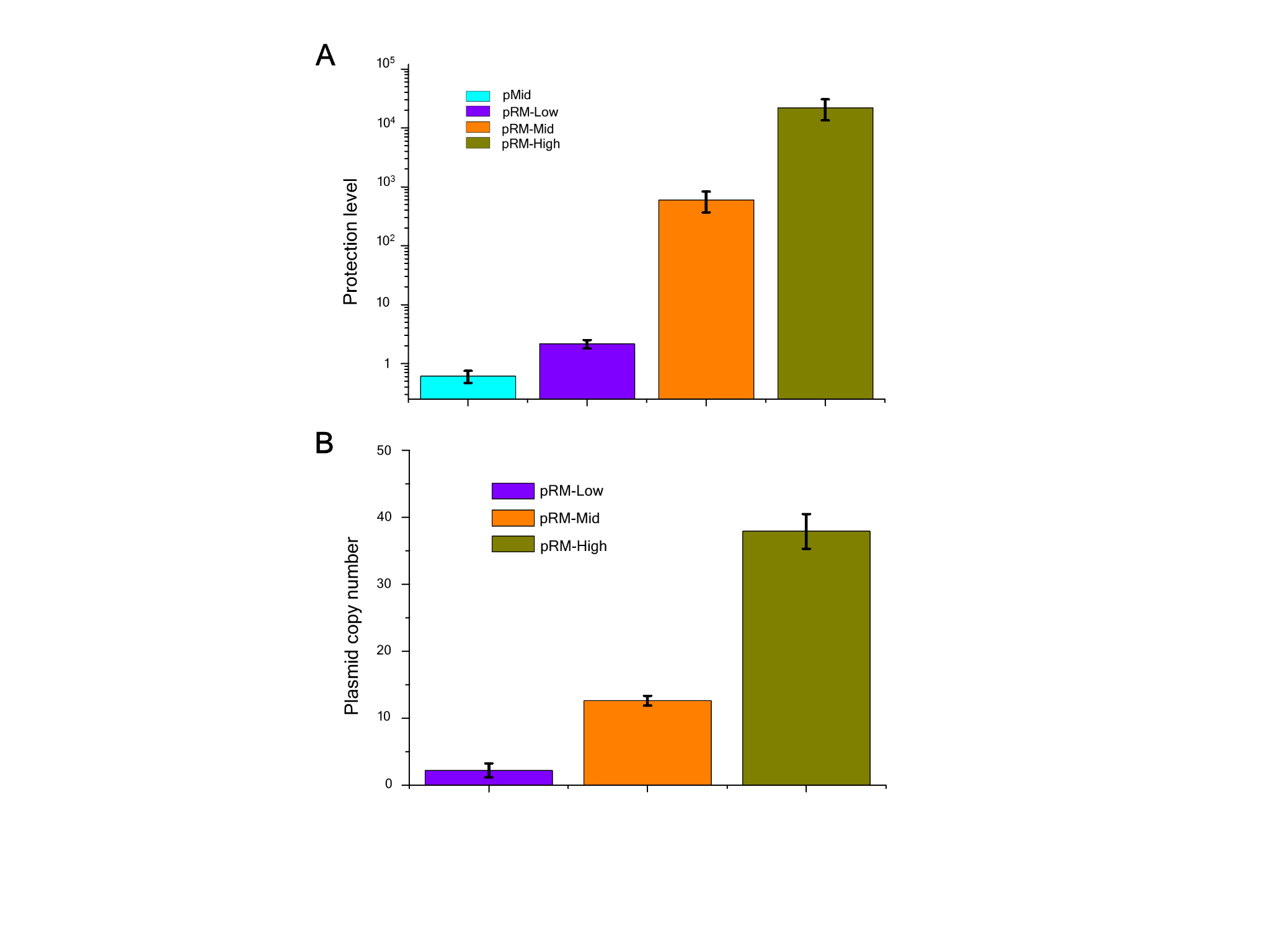


**Supplementary Figure S4.** Protection from phage λ_vir_ infection (**A**) and plasmid copy number (**B**) of MG1655 *seqA-mKO2 Δdam::KanR* cells carrying indicated plasmids. Protection levels and plasmid copy numbers were determined exactly as in Supplementary Figure S2 and S1, respectively.


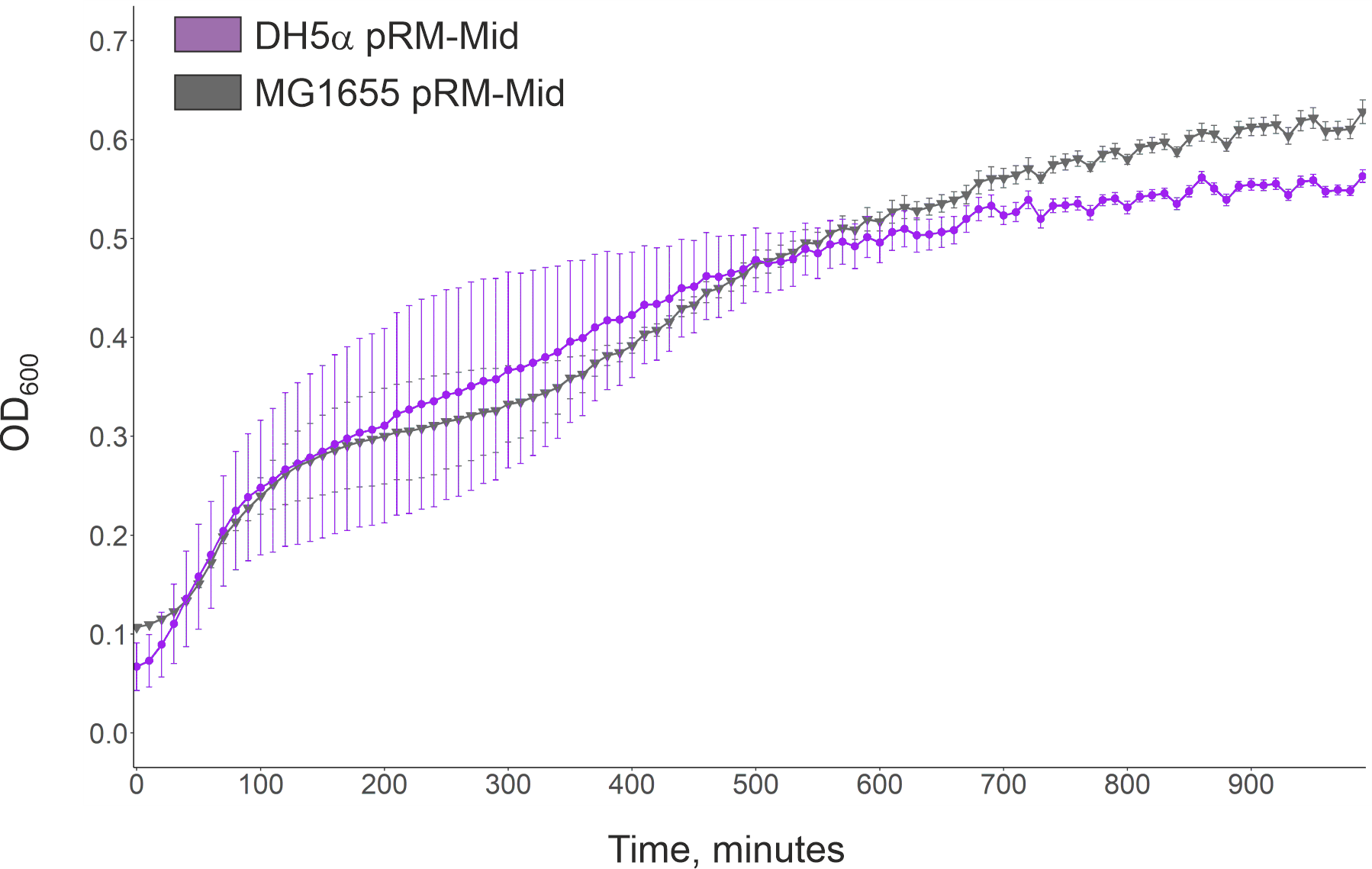


**Supplementary Figure S5.** Growth curves of cultures of DH5𝛼 (purple) and MG1655 *seqA-mKO2 Δdam::KanR* (grey) cells carrying the pRM-Mid plasmid. Error bars represent standard error of the mean.

**
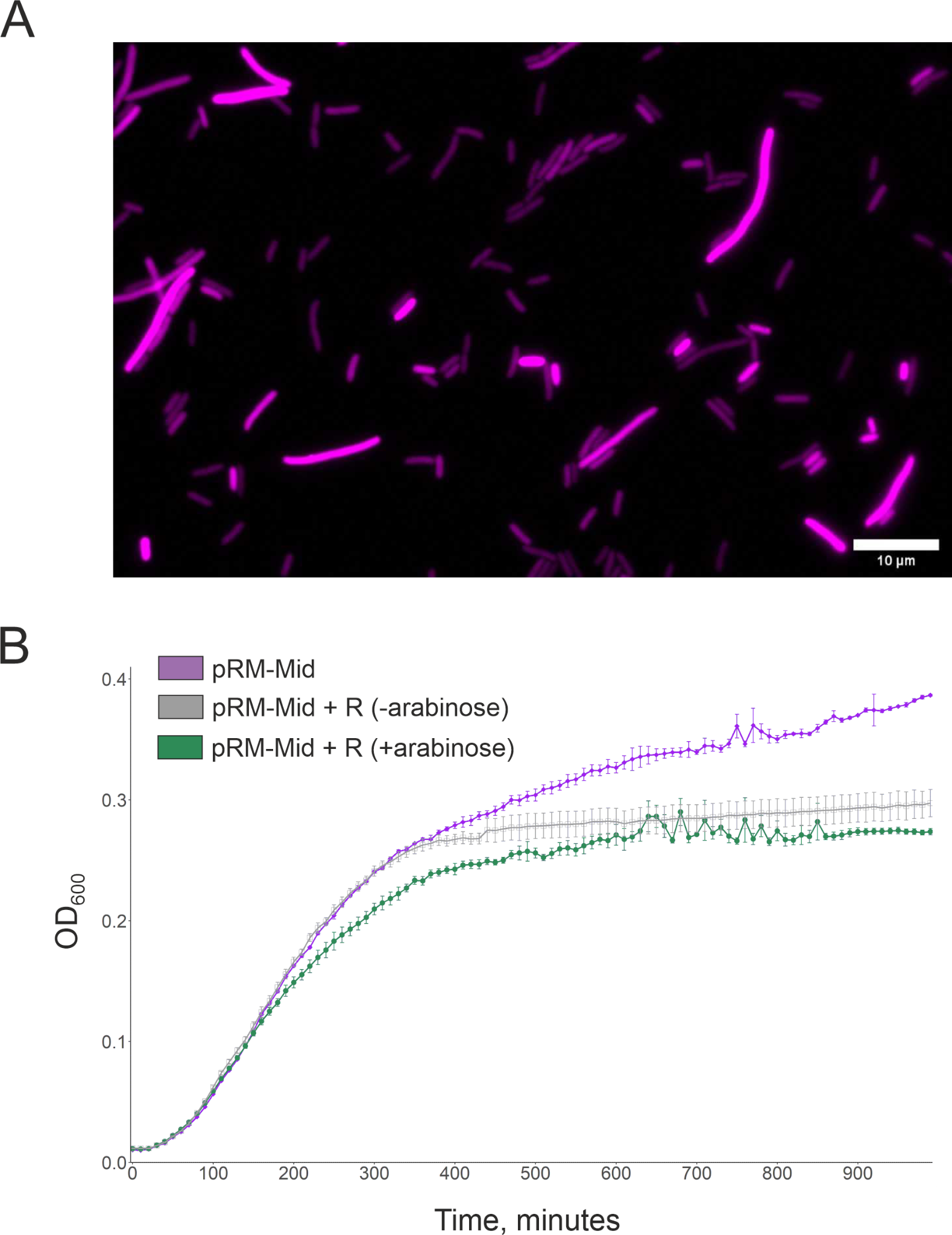
**

**Supplementary Figure S6.** The influence of additionally produced Esp1396I REase on morphology (**A**) and growth (**B**) of DH5a cells carrying the pRM-Mid plasmid.

(**A**) An representative image of the DH5𝛼 pRM-Mid+R cells in the mCherry (REase) channel. Live microscopy was performed on a sample collected 4 hours after the induction with 1 mM arabinose. Scale bar is 10 μm. Of 1238 cells analyzed, 83 (~6.7%) were extra bright of which ca. one third was also elongated.

(**B**) Growth of pRM-Mid (purple), or pRM-Mid+R DH5𝛼 cultures with (grey) or without (green) induction with 1 mM arabinose. Error bars represent standard errors of the mean.

**Supplementary Table S1.** Strains and plasmids used in this study.

| **Strain** | **Genotype** | **Source/**  **reference** |
| --- | --- | --- |
| **Bacterial strains** | | |
| DH5α | F– φ80*lacZ*ΔM15 Δ(*lacZYA*-*argF*)U169 *recA1* *endA1* hsdR17(*r_K–_, m_K+_*) *phoA* *supE44* λ– *thi*-*1 gyrA96 relA1* | (2) |
| Rosetta (DE3) | F– *ompT* *hsdS_B_(r_B-_ m_B-_*) *gal dcm* (DE3) pRARE (CmR) | (3) |
| LZ1663 | F–, λ–, *ilvG*-, *rfb*-50, *rph-1*, *seqA-mKO2 Δdam::KanR* | A K-12 MG1655 derivative described in Ref. 4 |
| **Phage** | | |
| λ_vir_ | A virulent mutant of bacteriophage lambda | Lab collection |
| **Plasmids** | | |
| pACYC184 | p15A ori, TetR, CmR | (5) |
| pLow (pSC101) | pSC101 ori, TetR | (6) |
| pRM-Low | Fluorescently labeled Esp1396I R-M system in pSC101 backbone with changed antibiotic resistance, AmpR | This work |
| pMid (pBR322) | pMB1 ori, TetR , AmpR | (7) |
| pRM-Mid | Fluorescently labeled Esp1396I R-M system in pBR322 backbone | This work |
| pACYC_R_fluo | pACYC184 with the *esp1396IR* gene cloned under the araBAD promoter, CmR | This work |
| pACYC_M_fluo | pACYC184 with the *esp1396IM* gene cloned under the araBAD promoter, CmR | This work |
| pHigh (pUC19) | pMB1 ori, AmpR | (8) |
| pRM-High | Fluorescently labeled Esp1396I R-M system cloned in the pUC19 backbone | (9) |
| **Primers** | | |
| R::mCherry_for | 5’-AAAGGTACCTTACTTGTACAGCTCGTCCATG | This work |
| R::mCherry_rev | 5’-AAAGGTACCATGACTAAAAATGTTAAGCTCCCACT | This work |
| pACYC_for | 5’-TAGCCGTCAAGTTGTCATAAACATCAGCGCTAGCGGA | This work |
| pACYC_rev | 5’-ATCCTGACGGATGGCCTTTTCCGGCGGTGCTTTTGC | This work |
| araBAD_for | 5’-AACGGCAAAAGCACCGCCGGAAAAGGCCATCCGTCAGGATG | This work |
| araBAD_rev | 5’-CACTCCGCTAGCGCTGATGTTTATGACAACTTGACGGCTACATCA | This work |
| M::Venus_for | 5’-AAACATATGTTACTTGTACAGCTCGTCCATGC | This work |
| M::Venus_rev | 5’-AAAGGTACCATGAACAGAGCTGAATCTTTTAAAAAAATC | This work |
| RM-Mid_for | 5’-AAGAATTCCATCAGAGCAGATTGTACTGAGAGTGC | This work |
| RM-Mid_rev | 5’-AAAAAGCTTGCATGCCTGCAGGTCGA | This work |
| pRM_for | 5’-CTTGTTACAGGAATTCAGATCCTTTGATCTTTTCTACGGG | This work |
| pRM_rev | 5’-ATCGCTGAATATTCCTCCCGGGCGGGTGCGCGTAATGAGACG | This work |
| pSC101_ori_F | 5’-CCCGGGAGGAATATTCAGCGAT | This work |
| pSC101_ori_R | 5’-GATCTGAATTCCTGTAACAAGTTGTCTCAGGTG | This work |
| **Primers for qPCR** | | |
| gyrA_for | 5’-CGGTCAACATTGAGGAAGAGC | This work |
| gyrA_rev | 5’-TACGTCACCAACGACACGG | This work |
| Esp1396I_meth_for | 5’-GCCTGAATATGAAGACGCAATAAG | This work |
| Esp1396I_meth_rev | 5’-CTGCTGGGTGCTTACCTTTA | This work |

**Supplementary references**

1. Livak,K.J. and Schmittgen,T.D. (2001) Analysis of relative gene expression data using real-time quantitative PCR and the 2(-Delta Delta C(T)) Method. *Methods San Diego Calif*, **25**, 402–408.

2. Taylor,R.G., Walker,D.C. and McInnes,R.R. (1993) E. coli host strains significantly affect the quality of small scale plasmid DNA preparations used for sequencing. *Nucleic Acids Res.*, **21**, 1677–1678.

3. Loyevsky,M., Mompoint,F., Yikilmaz,E., Altschul,S.F., Madden,T., Wootton,J.C., Kurantsin-Mills,J., Kassim,O.O., Gordeuk,V.R. and Rouault,T.A. (2003) Expression of a recombinant IRP-like Plasmodium falciparum protein that specifically binds putative plasmodial IREs. *Mol. Biochem. Parasitol.*, **126**, 231–238.

4. Trinh,J.T., Shao,Q., Guan,J. and Zeng,L. (2020) Emerging heterogeneous compartments by viruses in single bacterial cells. *Nat. Commun.*, **11**, 3813.

5. Chang,A.C. and Cohen,S.N. (1978) Construction and characterization of amplifiable multicopy DNA cloning vehicles derived from the P15A cryptic miniplasmid. *J. Bacteriol.*, **134**, 1141–1156.

6. Cohen,S.N., Chang,A.C.Y., Boyer,H.W. and Helling,R.B. (1973) Construction of Biologically Functional Bacterial Plasmids In Vitro. *Proc. Natl. Acad. Sci. U. S. A.*, **70**, 3240–3244.

7. Bolivar,F., Rodriguez,R.L., Greene,P.J., Betlach,M.C., Heyneker,H.L., Boyer,H.W., Crosa,J.H. and Falkow,S. (1977) Construction and characterization of new cloning vehicles. II. A multipurpose cloning system. *Gene*, **2**, 95–113.

8. Yanisch-Perron,C., Vieira,J. and Messing,J. (1985) Improved M13 phage cloning vectors and host strains: nucleotide sequences of the M13mpl8 and pUC19 vectors. *Gene*, **33**, 103–119.

9. Morozova,N., Sabantsev,A., Bogdanova,E., Fedorova,Y., Maikova,A., Vedyaykin,A., Rodic,A., Djordjevic,M., Khodorkovskii,M. and Severinov,K. (2016) Temporal dynamics of methyltransferase and restriction endonuclease accumulation in individual cells after introducing a restriction-modification system. *Nucleic Acids Res.*, **44**, 790–800.
